# The inter‐relationship of symptom severity and quality of life in 2055 patients with primary biliary cholangitis

**DOI:** 10.1111/apt.13794

**Published:** 2016-09-19

**Authors:** J. K Dyson, N. Wilkinson, L. Jopson, G. Mells, A. Bathgate, M. A. Heneghan, J. Neuberger, G. M. Hirschfield, S. J. Ducker, The UK‐PBC Consortium, R. Sandford, G. Alexander, D. Stocken, D. E. J. Jones

**Affiliations:** ^1^ Institute of Cellular MedicineNewcastle UniversityNewcastle‐upon‐TyneUK; ^2^ NIHR Newcastle Biomedical Research CentreNewcastle UniversityNewcastle‐upon‐TyneUK; ^3^ Institute of Health and SocietyNewcastle UniversityNewcastle‐upon‐TyneUK; ^4^ Department of HepatologyCambridge University Hospitals NHS Foundation TrustCambridgeUK; ^5^ Academic Department of Medical GeneticsUniversity of CambridgeCambridgeUK; ^6^ Scottish Liver Transplant UnitRoyal Infirmary of EdinburghEdinburghUK; ^7^ Institute of Liver StudiesKing's College Hospital NHS Foundation TrustLondonUK; ^8^ Centre for Liver ResearchNIHR Biomedical Research UnitUniversity of BirminghamBirminghamUK

## Abstract

**Background:**

Age at presentation with primary biliary cholangitis (PBC) is associated with differential response to ursodeoxycholic acid (UDCA) therapy. Younger‐presenting patients are less likely to respond to treatment and more likely to need transplant or die from the disease. PBC has a complex impact on quality of life (QoL), with systemic symptoms often having significant impact.

**Aim:**

To explain the impact of age at presentation on perceived QoL and the inter‐related symptoms which impact upon it.

**Methods:**

Using the UK‐PBC cohort, symptoms were assessed using the PBC‐40 and other validated tools. Data were available on 2055 patients.

**Results:**

Of the 1990 patients reporting a global PBC‐QoL score, 66% reported good/neutral scores and 34% reported poor scores. Each 10‐year increase in age at presentation was associated with a 14% decrease in risk of poor perceived QoL (OR = 0.86, 95% CI: 0.75–0.98, *P* < 0.05). All symptom domains were similarly age‐associated (*P* < 0.01). Social dysfunction was the symptom factor with the greatest impact on QoL. Median (interquartile range) PBC‐40 social scores for patients with good perceived QoL were 18 (14–23) compared with 34 (29–39) for those with poor QoL.

**Conclusion:**

The majority of patients with primary biliary cholangitis do not feel their QoL is impaired, although impairment is reported by a sizeable minority. Age at presentation is associated with impact on perceived QoL and the symptoms impairing it, with younger patients being more affected. Social dysfunction makes the greatest contribution to QoL impairment, and it should be targeted in trials aimed at improving life quality.

## Introduction

Primary biliary cholangitis [formerly known as primary biliary cirrhosis (PBC)] is an autoimmune chronic liver disease affecting approximately 18 000 patients in the UK.^1^, ^2^ The condition impacts on patients through progression to end‐stage liver disease with the development of the complications of cirrhosis and the need for liver transplantation.^3^ PBC patients also experience symptoms which can have an often significant impact on quality of life.^4^, ^5^ These symptoms include cholestatic pruritus and fatigue, the latter appearing to have a particular impact on life quality.^6^, ^7^, ^8^, ^9^

Therapy with the hydrophilic bile acid ursodeoxycholic acid (UDCA) is effective in the majority of patients; slowing disease progression and reducing risk of death and need for transplantation.^10^ Second‐line therapies are now emerging for the treatment of patients with inadequate response to UDCA.^11^, ^12^ It is becoming increasingly clear, however, that neither UDCA nor the emerging second‐line therapies for PBC are effective at treating symptoms. With the exception of anti‐pruritic agents, no therapies have yet been proven to improve patients’ quality of life. This potentially leads to a ‘therapy gap’ between disease prognosis, which improves with therapy, and life quality which does not.^12^ The current lack of effective therapy for systemic symptoms in PBC highlights the need for further research into the aetiology of symptoms and the development of novel therapies.^12^ Before we can effectively target quality of life impairment, however, it is essential that we understand the symptom factors that most impact on it, and develop plausible goals for therapy applicable in clinical trials and future practice.

Studies utilising the unique UK‐PBC national patient cohort, the largest prospective PBC cohort in the world,^13^, ^14^ have demonstrated that age is an important factor in the clinical expression of PBC in terms of disease severity, with patients presenting at younger ages being less likely to respond to UDCA.^15^ Furthermore, early studies using the cohort suggested that fatigue was worse in younger than older presenting patients suggesting that this group might represent one with specific unmet need.^7^ This gives rise to questions as to the impact of other symptoms in younger PBC patients and their contribution to quality of life impairment. In this study, we set out to use the UK‐PBC patient cohort data set to comprehensively explore the impact of symptoms on quality of life in younger patients and to model the complex relationships between age at presentation and reported symptoms. The goal is to identify drivers for poor perceived quality of life in younger patients, to characterise potentially modifiable symptoms which are under‐treated and could have impact in terms of improving quality of life and to begin to explore targets for effective therapy which will be essential for future treatment development.

## Materials and methods

### Patients and data collection

This study represents a further analysis of the UK‐PBC patient cohort dataset and builds on previous analyses.^5^, ^7^ Data were extracted from the UK‐PBC database based on patients enrolled into the cohort between 2008 and 2011. Approach to cohort development and data capture have been outlined previously.^5^, ^7^, ^13^, ^14^ Patients with ‘overlap syndromes’ are excluded from UK‐PBC. Quality of life and symptom data were collected during follow‐up using the patient‐completed PBC‐40 assessment tool,^16^ a median of 7 years after initial presentation with the condition. The PBC‐40 consists of 40 items in six independently validated domains relating to fatigue, emotional, social, cognitive function, general symptoms and itch. Each item is scored from 1 to 5, with higher scores denoting greater symptom severity. Other assessment tools completed were the Epworth Sleepiness Scale (ESS), Orthostatic Grading Scale (OGS) to assess autonomic dysfunction, Hospital Anxiety and Depression Scale (HADS)^5^; all of which have been successfully used in PBC previously and in which, again, higher scores denote worse symptoms. Participants were also asked to complete a global PBC‐related quality of life assessment; a question which asks patients how much they agree with the statement “PBC has affected my quality of life” (1: Strongly Disagree, 2: Disagree, 3: Neither Agree nor Disagree, 4: Agree, 5: Strongly Agree).

Liver biochemistry data were recorded for all patients and patients who had received UDCA treatment for a minimum of 1 year were assessed for biochemical response using the Paris 1 clinical response criteria (response is defined as bilirubin <1× the upper limit or normal (ULN), alanine transaminase (ALT) <2× ULN and alkaline phosphatase (AP) <3× ULN after a minimum of 1 year of UDCA therapy).^17^

### Statistical analyses

Descriptive statistics are presented as frequencies, *n* (%), and continuous covariates are presented as medians (interquartile range), due to underlying distributional assumptions. Dependent variables are analysed as (i) continuous symptom domain scores using linear regression modelling; (ii) global quality of life based on modelling poor (4 or 5) compared with better quality of life (1–3) using logistic regression modelling with 1 representing poor quality of life and 0 representing better quality of life; (iii) global quality of life based on modelling five categories of quality of life using ordinal regression. The ratio of risks of poor quality of life across levels of covariates is reported as an odds ratio (OR), where an odds ratio <1 indicates a reduced risk of poor quality of life.

Univariate regression models were developed for each covariate investigating nonlinear relationships using Fractional Polynomial Transformations.^18^ The best fitting model was selected based on the reduction in Akaike Information Criterion (AIC). Multivariable models were developed based on either full models of all covariates, or selecting variables for inclusion based on forward selection techniques. Forward selection models select factors which are independently explaining additional variability and hence significantly related to outcome. Where a forward selection method was used, covariates were included according to a 5% significance level. Continuous covariates are considered in the multivariable analysis according to their best fitting transformation. All multivariable models include adjustment for gender and albumin ratio (as an indicator of severity of liver disease), UDCA response and approximate disease duration (years), modelled based on a complete case dataset. UDCA response was characterised using the ‘Paris 1’ criteria (nonresponse being defined as any of ALT ratio >2 (ratio of the value reported to the ULN for the laboratory carrying out the measurement), AP ratio >3 or bilirubin ratio >1). Patients on UDCA for less than 1 year were excluded as were those with missing data. *R* ^2^ and pseudo *R* ^2^ (Nagelkerke *R* ^2^) have been reported for linear regression models and logistic regression models, respectively. Multi‐collinearity was measured using a variance inflation factor (VIF). Maximum likelihood factor analysis with a Varimax (orthogonal) rotation was conducted on the 10 quality of life domains. The aim was to investigate natural groupings of correlated domains and subsequently obtain interpretable loadings (weight) of each quality of life measure within a factor. The number of factors selected was based on eigenvalues and selected factors were used in a multivariable analysis.

ROC curves were calculated to explore suitable clinically relevant cut‐offs for patients under the age of 50 years of age for each quality of life domain below which patients are predicted to have a poor quality of life. The cut‐off was chosen to balance sensitivity and specificity.

## Results

The source data for this analysis were the UK‐PBC cohort symptoms dataset which has been described previously. This is a UK‐wide comprehensive cohort of PBC patients with detailed symptom and quality of life data.^5^, ^7^ Clinical data sets were available for a total of 2055 nontransplanted patients (although the UK‐PBC cohort includes transplanted patients these were excluded from this analysis) from 220 hospitals across the UK (Table 1). As previously reported, the majority (91%) of patients in the UK‐PBC cohort are female, with median age at presentation of 55 years (IQR 48–63) and median age at study entry of 65 years (IQR 57–72). The majority of patients (79%) were receiving UDCA therapy, however, 241 (14.8%) were excluded due to being on therapy <1 year and 231 (14.1%) patients were unable to have a UDCA response calculated because of missing data. None of the patients had had a liver transplant but nine were active on the transplant waiting list.

**Table 1:** Patient Characteristics at Study Entry. Note that all percentages are out of 2055 (the total number of patients) to show the levels of missing data apart from the two variables highlighted with a * which are out of 1629 (the number of people on UDCA)

Factor	Number of nonmissing data points in cohort of *N* = 2055 patients	*n* (% of *N*)	Median	IQR	Range
Gender (female)	2051	1858 (90.6)	–	–	–
Age at presentation (years)	1747		55	48–63	16–86
Age at study entry (years)	2053		65	57–72	21–91
Disease duration (years)	1747		7	4–12	0–37
Not awaiting liver transplant	2055	2046 (99.6)	–	–	–
On UDCA therapy	2055	1629 (79.3)	–	–	–
UDCA therapy length*					
<1 year	1448	241 (16.6)	–	–	–
1–5 years		578 (39.9)			
5–10 years		308 (21.3)			
≥10 years		321 (22.2)			
Response to treatment*					
UDCA Responder	1629	892 (54.8)	–	–	–
UDCA Nonresponder		265 (16.3)			
Excluded (on UDCA<1 year)		241 (14.8)			
Unknown		231 (14.1)			
PBC‐40 itch	1896		4	0–9	0–17
PBC‐40 symptoms	2022		16	12–20	5–33
PBC‐40 fatigue	2036		31	21–38	11–55
PBC‐40 cognitive	2011		12	6–18	6–30
PBC‐40 emotional	2007		7	5–11	3–15
PBC‐40 social	2025		23	16–31	10–50
ESS sleep	2045		7	4–11	0–24
OGS autonomic	2029		3	0–5	0–18
HADS anxiety	2044		7	3–10	0–21
HADS depression	2042		4	2–7	0–21
Global QoL (ordered)	1990		3	1–4	1–5
Global QoL (binary)					
Better	1990	1312 (65.9)	2	1–3	1–3
Poor		678 (34.1)	4	4–4.8	4–5
Global Health (ordered)	2021		3	3–4	1–5
Actual ALT level	1990		37	26–57	7–712
ALT ratio	1934		0.9	0.6–1.4	0.1–20.3
Actual ALP level	2018		183	126–322	33–2678
ALP ratio	1985		1.3	1.0–2.1	0.1–23.3
Actual Albumin	1887		41	38–44	18–80
Albumin ratio	1835		1.2	1.1–1.3	0.5–2.4
Actual Bilirubin	2004		9	7–13	2–168
Bilirubin ratio	1947		0.5	0.4–0.7	0.1–9.9

### Age as a predictor of global perceived quality of life

**Figure 1:**
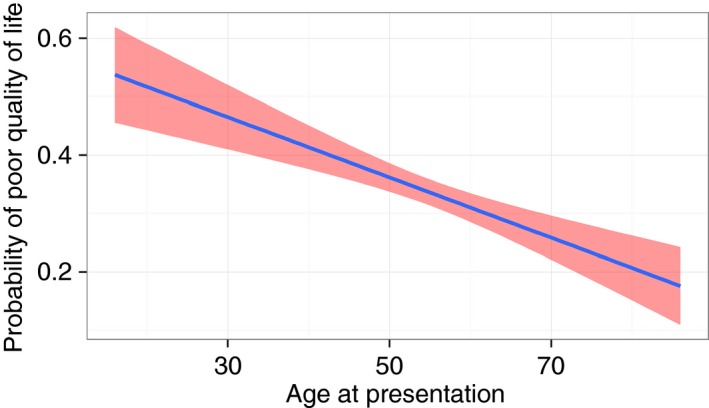
Predicted probability (blue line) with 95% CI (red shade) of poor perceived quality of life with increasing age at presentation.

**Figure 2:**
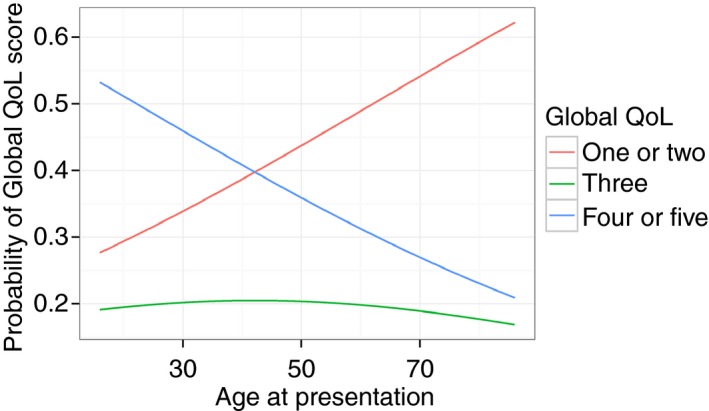
Probability of global quality of life scores with increasing age at presentation. The global quality of life is based on a question which asks patients how much patients agree with the statement “PBC has affected my quality of life” (1 or 2 represents disagreeing with the statement (strongly or weakly), 3: Neither Agreeing nor Disagreeing, 4 or 5 represents agreeing (weakly or strongly). The younger patients are at presentation the more likely they are to describe poor quality of life (4 or 5), the older they are the more likely they are to describe good quality of life (1 or 2).

A total of 1990 patients with a recorded age at presentation reported a global PBC‐quality of life score. Of these, 1312 (66%) patients reported good or neutral PBC‐related quality of life scores^1^, ^2^, ^3^ and 678 (34%) patients reported poor^4^, ^5^ quality of life scores; agreeing or strongly agreeing with the statement that PBC had impaired their quality of life (Table 1). Thus, although the majority of PBC patients do not feel their quality of life is impaired there is impairment seen in a sizeable minority. We did not set out, in the study protocol adopted, to assess non‐PBC related quality of life perception. A 10‐unit (10‐year) increase in age at presentation was associated with a 14% decreased risk of poor quality of life (OR = 0.86, 95% CI: 0.75–0.98, *P* < 0.05), after adjustment by gender, disease severity, UDCA response and disease duration (Table 2a and Figure 1). Using ordinal regression analysis, an increase in age at presentation is associated with an increase in the probability of being in the best quality of life group (‘1’) and a decrease in the probability of being in the worst quality of life group (‘5’) [Table 2b and Figure 2].

**Table 2:** Age at presentation and (a) overall relationship with overall perceived quality of life (good/poor)^a^ and (b) relationship with quality of life ordered from 1 (‘best’) to 5 (‘worst’). In each case model estimates are adjusted for gender, albumin ratio, UDCA response and disease duration^b^

(a)						
Outcome	Covariate	$\hat{\beta }$ (S.E.)	OR (CI)	*Z* value	*P* value	Pseudo *R* ^2^
Good vs. Poor Quality of life	Age at presentation (10 unit increase)	−0.16 (0.07)	0.86 (0.75–0.98)	−2.30	0.02	0.03
	Male	−0.04 (0.23)	0.97 (0.61–1.51)	−0.15	0.88	
	Albumin ratio	−0.56 (0.38)	0.57 (0.27–1.20)	−1.46	0.15	
	UDCA responder	−0.28 (0.16)	0.76 (0.56–1.04)	−1.75	0.08	
	Disease duration	0.02 (0.01)	1.02 (1.00–1.05)	1.97	0.05	

(b)					
	${\hat{\beta }}_{i}$ (S.E.)	OR (CI)	*t* value	*P* value	Pseudo *R* ^2^
Age at presentation (10 units)	−0.16 (0.06)	0.85 (0.76, 0.96)	−2.75	0.006	0.04
Male	−0.25 (0.20)	0.78 (0.53, 1.15)	−1.24	0.21	
Albumin ratio	−0.79 (0.32)	0.45 (0.24, 0.85)	−2.48	0.01	
UDCA responder	−0.36 (0.14)	0.70 (0.53, 0.91)	−2.65	0.008	
Disease duration	0.02 (0.01)	1.02 (1.00, 1.04)	1.82	0.07	
State 1–2	−3.17 (0.52)		−6.06	*P* < 0.001	
State 2–3	−2.19 (0.52)		−4.22	*P* < 0.001	
State 3–4	−1.32 (0.52)		−2.56	0.01	
State 4–5	0.40 (0.52)		0.77	0.44	

a In this analysis quality of life outcome is modelled on 1015 patients; 654 reporting good and 361 reporting poor quality of life.

b In this analysis quality of life outcome is modelled on 1015 patients; 233 patients reporting 1 (best quality of life), 211 patients reporting 2, 210 patients reporting 3, 267 patients reporting 4 and 94 patients reporting 5 (worst). Ordinal regression allows the ordinal nature of the global quality of life outcome (scored 1 ‘best’ to 5 ‘worst’) to be retained. The underlying assumption of proportional odds was confirmed graphically and confirming no significant difference in the likelihood ratio test comparing a proportional odds model to a multinomial‐logit (nonproportional odds) model. This analysis confirms the increasing probability of ‘better’ global quality of life impairment scores with increasing age at presentation and confirms a 10‐unit increase in age at presentation to be associated with a 15% reduction in risk of poorer quality of life (OR = 0.85, 95% CI: 0.76–0.96, *P* < 0.01).

### Age as a predictor of individual symptom severity

There were minimal missing data for the patient reported symptom scores; PBC‐40, HADS anxiety, HADS depression, ESS sleep and OGS autonomic scales. Symptom severity was strongly associated with age at presentation for all symptoms (Table 3). The relationship between symptom severity and age at presentation was linear for all the PBC‐40 domains except itch (which was best explained with a first‐degree fractional polynomial [age^−0.5^]). The association between age at presentation and sleep and autonomic scores were both best explained with the same second‐degree fractional polynomial [age^3^ + age^3^log (age)]. For all domains and symptom scores, increasing age at presentation is associated with decreased PBC‐40 domain score. However, the amount of variability explained in each is low (*R* ^2^ < 0.063) indicating that the variability in each domain cannot be explained by age alone.

**Table 3:** Age at presentation (‘Age’) and relationship with each quality of life domain adjusted for gender, albumin ratio, UDCA response and disease duration

	${\hat{\beta }}_{i}$ (S.E.)	*t* value	*P* value	*R* ^2^
PBC‐40 itch				
Age^−0.5^ (10 unit increase)	16.13 (3.09)	5.21	<0.001	0.06
Male	−0.95 (0.50)	−1.91	0.06	
Albumin ratio	−1.06 (0.81)	−1.31	0.19	
UDCA response	−1.10 (0.35)	−3.16	<0.01	
Duration	−0.01 (0.03)	−0.40	0.69	
PBC‐40 symptoms				
Age (10 unit increase)	−0.78 (0.17)	−4.63	<0.001	0.06
Male	−2.88 (0.58)	−4.98	<0.001	
Albumin ratio	−1.56 (0.95)	−1.65	0.10	
UDCA response	0.36 (0.41)	0.88	0.38	
Duration	0.06 (0.03)	1.82	0.07	
PBC‐40 fatigue				
Age (10 unit increase)	−1.49 (0.34)	−4.34	<0.001	0.05
Male	−4.30 (1.17)	−3.67	<0.001	
(Albumin ratio)^−2^	5.92 (1.59)	3.71	<0.001	
UDCA response	0.03 (0.83)	0.03	0.97	
Duration	0.04 (0.06)	0.65	0.52	
PBC‐40 cognitive				
Age (10 unit increase)	−0.71 (0.20)	−3.61	<0.001	0.02
Male	−1.03 (0.68)	−1.51	0.13	
Albumin ratio	−1.65 (1.10)	−1.50	0.13	
UDCA response	−0.46 (0.48)	−0.95	0.34	
Duration	0.01 (0.04)	0.30	0.76	
PBC‐40 emotional				
Age (10 unit increase)	−0.66 (0.11)	−6.16	<0.001	0.06
Male	−0.96 (0.37)	−2.62	<0.01	
Albumin ratio	−1.92 (0.60)	−3.21	<0.01	
UDCA response	0.01 (0.26)	0.02	0.98	
Duration	−0.02 (0.02)	−1.20	0.23	
PBC‐40 social				
Age (10 unit increase)	−1.24 (0.30)	−4.17	<0.001	0.04
Male	−1.42 (1.02)	−1.40	0.16	
(Albumin ratio)^−2^	4.48 (1.39)	3.23	<0.01	
UDCA response	−1.13 (0.72)	−1.57	0.12	
Duration	0.07 (0.05)	1.40	0.16	
ESS sleep				
Age^3^ (10 unit increase)	−0.10 (0.03)	4.89	<0.001	0.03
Age^3^log(age) (10 unit increase)	0.04 (0.01)			
Male	0.38 (0.54)	0.72	0.47	
Albumin ratio	−0.74 (0.87)	−0.85	0.39	
UDCA response	−0.58 (0.38)	−1.53	0.13	
Duration	−0.03 (0.03)	−1.16	0.25	
OGS autonomic				
Age^3^ (10 unit increase)	−0.05 (0.02)	3.05	<0.01	0.02
Age^3^log(age) (10 unit increase)	0.03 (0.01)			
Male	−0.94 (0.36)	−2.63	<0.01	
Albumin ratio	−0.34 (0.58)	−0.58	0.56	
UDCA response	0.16 (0.25)	0.62	0.53	
Duration	0.02 (0.02)	1.09	0.28	
HADS anxiety				
Age (10 unit increase)	−0.64 (0.14)	−4.59	<0.001	0.04
Male	−1.94 (0.48)	−4.06	<0.001	
Albumin ratio	−0.27 (0.78)	−0.35	0.72	
UDCA response	0.31 (0.34)	0.92	0.36	
Duration	−0.03 (0.02)	−1.05	0.29	
HADS depression				
Age (10 unit increase)	−0.39 (0.12)	−3.32	<0.001	0.03
Male	−0.37 (0.40)	−0.90	0.37	
(Albumin ratio)^−2^	1.78 (0.55)	3.23	<0.01	
UDCA response	−0.37 (0.29)	−1.30	0.19	
Duration	0.01 (0.02)	0.47	0.64	

### Symptom predictors of global quality of life

In keeping with previous analyses, the global quality of life score was found to be strongly associated with all symptom severity scores on univariate analysis (Table S1). The relationship between global quality of life score and symptom severity was linear for each of the PBC‐40 domains. In contrast, sleep and depression were both best explained by second‐degree fractional polynomials. Estimates for all individual symptom domain scores with global quality of life score were positive indicating, unsurprisingly, that an increased score results in an increased risk of a poor quality of life. For example, a 1‐unit increase in HADS anxiety score was associated with a 21% increased risk of a poor quality of life (OR = 1.21, 95% CI: 1.18–1.24). A forward selection model, based on a total of 1006 patients with 360 (36%) reporting poor quality of life showed that following adjustment for the effects of age at presentation, as well as gender, albumin level (as a marker for advanced disease), disease duration, and UDCA response, identified the symptom domains of social, fatigue, anxiety and depression as important predictors of poor perceived quality of life (in descending order) [Table 4]. The single most important predictor of poor quality of life is social functioning with a 27% increased risk of being in the ‘poor’ quality of life group with a unit increase in social functioning score (OR = 1.27, 95% CI 1.22–1.33). Note that, the domains are correlated so this model suffers from multi‐collinearity. This may explain why the coefficient for anxiety is negative which contradicts the findings in Table S1.

**Table 4:** Multivariable analysis of symptom scores adjusted by age at presentation, gender, albumin ratio, UDCA response and disease duration as predictors of global quality of life

	${\hat{\beta }}_{i}$ (S.E.)	OR (CI)	*z* value	*P* value	VIF	Pseudo *R* ^2^
PBC‐40 social	0.24 (0.02)	1.27 (1.22–1.33)	11.05	<0.001	1.38	0.69
PBC‐40 fatigue	0.07 (0.02)	1.07 (1.03–1.10)	4.03	<0.001	1.29	
HADS anxiety	−0.08 (0.03)	0.92 (0.87–0.98)	−2.75	<0.01	1.43	
HADS depression (depression + 1)^−0.5^	−2.49 (1.27)	0.08 (0.01–0.95)	−1.96	0.05	1.47	
HADS depression (depression + 1)^3^	−2.19 × 10^−6^ (2.01 × 10)^−4^	1.00 (1.00–1.00)	−0.01	0.99	
Male	0.53 (0.40)	1.70 (0.77–3.74)	1.33	0.18	1.09	
Duration	0.02 (0.02)	1.02 (0.99–1.06)	1.27	0.20	1.11	
UDCA Response	−0.26 (0.25)	0.77 (0.47,1.26)	−1.04	0.30	1.08	
Albumin ratio	0.12 (0.61)	1.13 (0.34–3.68)	0.20	0.84	1.47	
Age at presentation	0.003 (0.11)	1.00 (0.81–1.24)	0.03	0.98	1.18	

Quality of life outcome is modelled on 1006 patients; 646 reporting good and 360 reporting poor quality of life.

To investigate and group correlated symptom scores, and address multi‐collinearity, a maximum likelihood factor analysis was performed based on a single factor as suggested by the eigenvalues (Table S2a). A single factor explained 54% of the total variability dominated by social, fatigue, depression, and emotional domains. The analysis was repeated based on two factors to investigate robustness of the conclusions. A two factor analysis explains 57% of total variability in the quality of life domains, of which the first factor explains 29% of this variability (Table S2b). The same four domains (social, fatigue, depression and emotional), as well as anxiety, dominate the analysis as emotional, depression, social and anxiety dominate the first factor and fatigue dominates the second factor. Including the second factor explains only 3% more variability, confirming the single factor analysis to be more appropriate. The relationship between the quality of life domains and overall quality of life can be explored using the factor in Table S2a and fitting a logistic regression model adjusted for gender, albumin, UDCA response, disease duration and age at presentation (Table S3).

**Figure 3:**
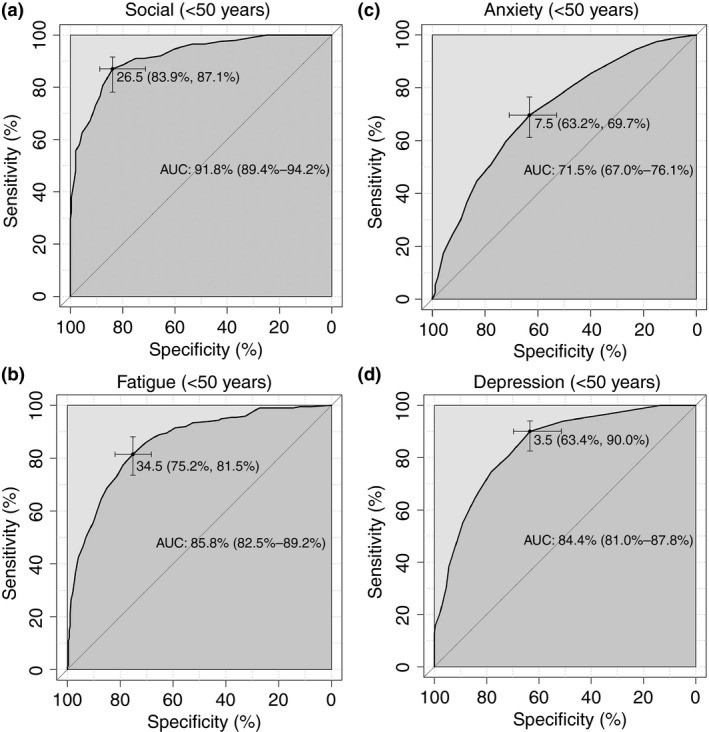
ROC curves of (a) PBC‐40 social domain, (b) PBC‐40 fatigue domain, (c) HADS anxiety, (d) HADS depression, to predict poor quality of life in the 493 patients presenting under the age of 50. The symptoms analysed are those that are predictive of poor perceived quality of life (Table 4).

The median values for the symptom severity scores for the good and poor quality of life group across the whole cohort are given in Table 5a. Given the symptom burden, and impact of quality of life impairment on the younger‐presenting patient we set out to identify cut‐off values for the symptom domains in the 493 patients in the cohort who presented under 50 years of age that could be explored as targets for therapy interventions in the future (Table 5b and Figure 3). Unsurprisingly, social dysfunction symptoms were most predictive of poor life quality in younger patients with an area under the curve of 91.8 (89.4–94.2) and an optimal cut‐off of less than 26.5 indicating poor quality of life for the PBC‐40 social domain.

**Table 5:** Symptom scores by good/poor quality of life (a) Summary statistics across the whole cohort for the symptom domain scores in PBC patients with good and poor quality of life. (b) ROC analysis in the 493 patients in the cohort presenting under the age of 50

(a)						
	Good quality of life			Poor quality of life		
	Median	IQR	Range	Median	IQR	Range
PBC‐40 social	18	14–23	10–41	34	29–39	15–50
PBC‐40 fatigue	26	16–32	11–55	40	34–45	11–55
HADS anxiety	5	3–8	0–20	9	6–12	0–21
HADS depression	3	1–5	0–13	8	5–11	1–21
PBC‐40 emotional	6	4–8	3–15	11	8–13	3–15
PBC‐40 cognitive	9.5	6–14	6–30	18	14–21	6–30
PBC‐40 itch	3	0–7	0–17	8	4–11	0–15
PBC‐40 symptoms	14	10–18	5–32	19	16–23	6–33
OGS autonomic	1	0–4	0–18	5	2–7	0–18
ESS sleep	6	3–9	0–24	10	6–14	0–24

(b)				
	Cut‐off	Specificity	Sensitivity	AUC (95% CI)
PBC‐40 social	26.5	83.9	87.1	91.8 (89.4–94.2)
PBC‐40 fatigue	34.5	75.2	81.5	85.8 (82.5–89.2)
HADS anxiety	7.5	63.2	69.7	71.5 (67.0–76.1)
HADS depression	3.5	63.4	90.0	84.4 (81.0–87.8)

## Discussion

The UK‐PBC patient cohort is the largest fully characterised PBC patient cohort in the world. Using this dataset, we have previously demonstrated that age of onset of PBC is associated with specific disease characteristics, with patients presenting at younger ages having a significantly lower likelihood of responding to therapy with UDCA.^5^, ^7^ Our earlier analysis also suggested a potential association between age at presentation and severity of two of the individual symptoms of PBC, namely fatigue and pruritus. This raises important questions relating to the broader phenotype of disease in younger patients and their treatment needs. The inter‐relationships of symptom sets, and their linked impacts on perceived quality of life mean that a more integrated approach to modelling symptom sets is appropriate and this is the approach undertaken in the novel analysis presented here. The unique UK‐PBC data set allows us to begin to understand the complex impact of symptoms on life quality in younger patients. This will allow us to set benchmark values for key symptom severity if we are to improve quality of life, and inform future studies to identify the key targets for therapeutic intervention.

In this study, we have demonstrated that both overall perception of PBC‐related quality of life (we did not set out to assess non‐PBC‐related quality of life), and the severity of all individual symptoms, is, as is the case with UDCA response, strongly related to the age of onset of disease, with younger presenting patients experiencing the greatest impact. Importantly, this remains the case after correcting for disease duration, UDCA response and disease severity. Taken together, therefore, current evidence cumulatively shows that younger‐presenting patients are less likely to respond to therapy, are more likely to need liver transplantation at some point in their disease course and have more severe symptoms. We also demonstrate a hierarchy in terms of the impact of individual symptoms on overall perceived life quality, with the greatest impact coming from symptoms of social isolation; a finding which opens up new opportunities for treatment targeting for quality of life improvement in PBC.

We have previously reported, and others have confirmed, that the likelihood of response to UDCA therapy in PBC is age‐related, with patients presenting at younger ages being less likely to respond.^7^ Taken together with the current observation of greater symptom impact and quality of life impairment in younger patients, this raises the possibility that the biology and/or natural history of PBC may differ between different patient groups, with younger‐presenting patients having a more aggressive or materially different form of the disease. While our findings may reflect ascertainment bias (diagnosis of PBC in younger patients, a group not typically thought of as being at risk of PBC, being more likely in symptomatic patients who undergo clinical assessment for their symptoms), the impact of these symptoms on life quality is real for patients. The overall perception of PBC‐related quality of life impairment is also strongly linked to age at presentation, with younger patients perceiving the greatest impact on life quality. This finding further challenges the view that PBC is a relatively benign condition of typically older people with limited clinical impact, and emphasises the need to specifically consider and address the complex disease impact in younger patients. Although we do not hold data on comorbidity and inter‐current drug therapy numbers, both these parameters characteristically increase with increasing patient age, suggesting that they are unlikely to be confounders in an analysis which shows increased symptom load at younger ages. This study did not include a comparator disease group as there are no comparable comprehensive liver disease patient cohorts. However, work in inflammatory bowel disease (IBD) found that diminished IBD‐quality of life was predicted by younger age and social interaction variables were associated with IBD‐quality of life.^19^ In contrast, fatigue associated with renal transplantation has a greater symptomatic impact in older patients.^20^

The relationship between symptoms and impact on quality of life is complex, with personality and coping skills acting as important modifying factors in individual patients. These additional elements in the conversion of a specific symptom into the expression of quality of life impairment may also go some way to explaining why symptom impact is seemingly different in different populations with fatigue, for example, being less of an issue in some populations than in the UK patient group. The advent of very large data sets, such as in UK‐PBC, allow us to smooth out these individual aspects and begin to look for over‐arching trends (including, now, the impact of age). Although understanding life quality requires a complex approach (exploring complex aspects in an individual way; the rationale behind the development of the domain‐based PBC‐40 quality of life measure), taking an over‐arching view of patient perception is also valuable and allows us to gauge the perception of patients as to their summary health status. Ultimately, patient perception is critical for effective management as the inter‐relationship between this and the individual underpinning symptoms allows identification of the symptoms with the greatest impact on perception of life quality. These symptoms can then be prioritised for clinical intervention. One important potential explanation for enhanced symptom impact in younger patients may be age‐related differences in expectation of likelihood of chronic disease, personal coping skills and support networks. In simple terms, younger PBC patients (and those with other chronic diseases showing the same symptom impact pattern) are less prepared for chronic disease (it is not within their reasonable expectation as to what might happen to them), and cope less well, are less likely to have relevant support networks (peer group will also be young people typically without chronic disease) and the expectation of normal life (and thus the gap between expectation and reality will be greater). Further work is needed to explore these issues. Understanding the complex psychosocial factors underpinning age‐related quality of life impairment will be essential, if we are to support patients better as it is unlikely that purely pharmaceutical approaches will be effective for such a complex problem.

Our most striking finding is that the symptom domain with the greatest impact on perceived quality of life was the PBC‐40 social domain; a domain focused around symptoms linked to social isolation. The greatest impact is again seen in younger patients. This may be as a consequence of the perceived ‘loss’ associated with the disease; limiting the opportunities to have a normal age‐appropriate social life. This finding gives rise to important questions and therapeutic opportunities. Social functioning is a modifiable symptom and this research suggests that addressing and treating this single aspect could improve global quality of life significantly. Anecdotal reports from patients give insight into the reasons for social isolation; ranging from fatigue limiting capacity^21^ and desire to undertake social activity, to self‐consciousness relating to itching secondary to pruritus.^22^ Reports of coping strategies adopted by fatigued patients often highlight reducing work and other perceived energy‐saving approaches. While offering the potential for short‐term gain in terms of fatigue, such approaches clearly have the potential to further narrow social networks, exacerbating social isolation and potentially worsening quality of life. The opportunity related to our finding is to develop novel approaches to support social structures for symptomatic patients with the potential for a nonpharmaceutical improvement in quality of life. Such approaches could range from simple counselling to alert patients to the potential for social isolation,^23^ to the development of support groups (PBC research and support groups may have an important role to play), to the development of newer digital approaches to social networking through social media (which may be of particular value to younger patients). We have used ROC analysis to identify optimal cut‐offs for the symptoms that predict impaired quality of life in our cohort. The value of these as targets to be achieved in therapeutic intervention studies should be explored.

The other key factors associated with poor perceived quality of life in younger patients are fatigue, anxiety and depression. The association with fatigue is unsurprising given previous study findings and the identification in patient surveys of fatigue as the biggest extant symptom problem in PBC. The impact of fatigue on life quality means that identification of the causes of fatigue and approaches to its treatment should be research priorities. Care should be taken, however, in managing fatigue to balance the potentially conflicting issues of fatigue and social isolation. The individual associations of fatigue and depression with poor PBC‐quality of life would further suggest that fatigue in PBC is not caused by depression. Depression and anxiety are important factors in patient experience and should be explored in patients with poor PBC‐quality of life. These aspects may be related to fear of the future and ability to cope, uncertainty as to disease prognosis and frustration at limitations to life quality. It is striking that in contrast to the symptom domains, advanced liver disease (assessed through the surrogate marker of serum albumin) does not associate with perceived PBC‐quality of life. This is likely to reflect the fact that the majority of patients with PBC do not have advanced disease at any one time, and that the symptom sets that do impact on quality of life are not severity‐associated.

This study sheds new light on the complex issues underpinning the poor quality of life reported by younger PBC patients. Our findings emphasise the complexity of the factors linked to poor quality of life and suggest that a holistic approach to them is essential. While specifically targeting fatigue is likely to pay dividends, there are currently no therapies able to do that. In contrast, a more sociological approach targeting social isolation and the depression and anxiety which may accompany it are very viable approaches. The benefits of developing coping strategies informed by our understanding of the impact of social isolation should be actively explored.

## Authorship

*Guarantor of the article*: Professor David Jones.

*Author contributions*: JKD, LJ and DEJJ have interpreted the data and written the manuscript. NW and DS have analysed and interpreted the data. GM coordinated data acquisition and made substantial contributions to the conception and design of the study and reviewed the manuscript. AB, MH, JN, GH, SJD, RS, GA have developed the UK‐PBC cohort and reviewed the manuscript. All authors agree to be accountable for all aspects of the work in ensuring that questions related to the accuracy or integrity of any part of the article are appropriately investigated and resolved.

All authors approved the final version of the article, including the authorship list.

## Supporting information


**Table S1.** Univariate analysis of age and symptom scores as predictors of global quality of life (good, poor) using logistic regression (poorer quality of life compared with better quality of life).
**Table S2a.** Results showing the factor loadings of a factor analysis with a single factor and the percentage of variance in the response explained by the factor.
**Table S2b** ***.*** Results showing the factor loadings of a factor analysis with two factors and the percentage of variance in the response explained by the factors.
**Table S3.** Logistic regression including single factor loadings as a covariate adjusted by gender, albumin ratio, UDCA response, disease duration and age at presentation as predictors of global quality of life (good, poor).

**Data S1.** The UK‐PBC Consortium
