# Supplementary material for: The inter‐relationship of symptom severity and quality of life in 2055 patients with primary biliary cholangitis
Source: Aliment Pharmacol Ther. 2016 Sep 19;44(10):1039–50. doi: 10.1111/apt.13794 (PMC5082554; doi:10.1111/apt.13794)
Supplement: Supplementary file 1 — Table S1. Univariate analysis of age and symptom scores as predictors of global quality of life (good, poor) using logistic regression (poorer quality of life compared with better quality of life). Table S2a. Results showing the factor loadings of a factor analysis with a single factor and the percentage of variance in the response explained by the factor. Table S2b . Results showing the factor loadings of a factor analysis with two factors and the percentage of variance in the response explained by the factors. Table S3. Logistic regression including single factor loadings as a covariate adjusted by gender, albumin ratio, UDCA response, disease duration and age at presentation as predictors of global quality of life (good, poor). [file APT-44-1039-s001.docx]

***Supplementary Table 1*:** Univariate analysis of age and symptom scores as predictors of global quality of life (good, poor) using logistic regression (poorer quality of life compared with better quality of life).

|  | Transformation | Beta (se) | OR (CI) | z value | p value | Pseudo R^2^ |
| --- | --- | --- | --- | --- | --- | --- |
| Age at diagnosis (10 units) | Linear | -0.23 (0.05) | 0.79 (0.72,0.87) | -4.94 | <0.001 | 0.02 |
| PBC-40 Itch | Linear | 0.15 (0.01) | 1.16 (1.13,1.19) | 12.65 | <0.001 | 0.13 |
| PBC-40 Symptoms | Linear | 0.20 (0.01) | 1.22 (1.19,1.24) | 17.54 | <0.001 | 0.26 |
| PBC-40 Fatigue | Linear | 0.17 (0.01) | 1.19 (1.17,1.21) | 21.21 | <0.001 | 0.49 |
| PBC-40 Cognitive | Linear | 0.19 (0.01) | 1.21 (1.19,1.24) | 19.27 | <0.001 | 0.31 |
| PBC-40 Emotional | Linear | 0.41 (0.02) | 1.51 (1.46,1.57) | 21.59 | <0.001 | 0.41 |
| PBC-40 Social | Linear | 0.28 (0.01) | 1.32 (1.29,1.36) | 22.61 | <0.001 | 0.67 |
| ESS sleep | 2 degree FP  (sleep+1)^2^  (sleep+1)^2^log(sleep+1) | 0.04 (0.01)  -0.011 (0.002) | NA | 14.68 | <0.001 | 0.16 |
| OGS autonomic | Linear | 0.25 (0.02) | 1.28 (1.24-1.32) | 15.21 | <0.001 | 0.18 |
| HADS anxiety | Linear | 0.19 (0.01) | 1.21 (1.18,1.24) | 15.75 | <0.001 | 0.19 |
| HADS depression | 2 degree FP  (depression+1)^-0.5^  (depression+1)^3^ | -8.36 (0.77)  5.83x10^-4^ (1.27x10^-4^) | NA | 19.77 | <0.001 | 0.44 |
| Albumin ratio | Linear | -0.37 (0.29) | 0.69 (0.39,1.23) | -1.25 | 0.21 | 0.001 |

NA=not applicable

***Supplementary Data Table 2a)*** Results showing the factor loadings of a factor analysis with a single factor and the percentage of variance in the response explained by the factor

|  | Factor 1 |
| --- | --- |
| PBC-40 social | 0.88 |
| PBC-40 fatigue | 0.87 |
| HADS depression | 0.83 |
| PBC-40 emotional | 0.81 |
| PBC-40 cognitive | 0.77 |
| PBC-40 symptoms | 0.71 |
| HADS anxiety | 0.71 |
| OGS autonomic | 0.59 |
| ESS sleep | 0.55 |
| PBC-40 itch | 0.47 |
| % variance explained | 54% |

***Supplementary Data Table 2b)*** Results showing the factor loadings of a factor analysis with two factors and the percentage of variance in the response explained by the factors

|  | Factor 1 | Factor 2 |
| --- | --- | --- |
| PBC-40 emotional | 0.75 | 0.40 |
| HADS depression | 0.70 | 0.47 |
| PBC-40 social | 0.68 | 0.56 |
| HADS anxiety | 0.68 | 0.31 |
| PBC-40 cognitive | 0.51 | 0.58 |
| PBC-40 fatigue | 0.47 | 0.79 |
| PBC-40 symptoms | 0.38 | 0.63 |
| OGS autonomic | 0.37 | 0.46 |
| ESS sleep | 0.24 | 0.55 |
| PBC-40 itch | 0.23 | 0.44 |
| % variance explained | **29.0%** | **57.0%** |

**Supplementary Table 3:**  Logistic regression including single factor loadings as a covariate adjusted by gender, albumin ratio, UDCA response, disease duration and age at presentation as predictors of global quality of life (good, poor)

|  | $\hat{\boldsymbol{\beta}}$ (s.e) | OR (CI) | Z value | VIF | P value | Pseudo R^2^ |
| --- | --- | --- | --- | --- | --- | --- |
| (Intercept) | -2.32 (0.91) | 0.10 (0.02, 0.58) | -2.56 |  | < 0.05 | 0.60 |
| Factor One | 2.61 (0.17) | 13.60 (9.82,19.43) | 15.00 | 1.07 | < 0.001 |  |
| Albumin | 0.20 (0.56) | 1.22 (0.40, 3.62) | 0.35 | 1.02 | 0.72 |  |
| Male | 0.70 (0.37) | 2.02 (0.98, 4.12) | 1.92 | 1.06 | 0.06 |  |
| UDCA Response | -0.31 (0.23) | 0.73 (0.46, 1.15) | -1.35 | 1.07 | 0.18 |  |
| Disease duration | 0.03 (0.02) | 1.03 (1.00, 1.07) | 1.67 | 1.12 | 0.10 |  |
| Age at presentation | 0.02 (0.01) | 1.02 (1.00, 1.04) | 1.59 | 1.20 | 0.11 |  |

This table shows that increasing the factor one score dramatically increases the risk of a poor quality of life (OR=13.11, 95% CI 9.51, 18.61). Note that this OR is more difficult to interpret as the domain scores are standardised before analysis. In this model, the problem of multi-collinearity is solved as the variance inflation factor (VIF) score for the factor is much closer to one than the VIF scores for the multivariable model in **T*able 4***. Anxiety, as well as all other domains, are represented positively in the factor, therefore increasing any domain is related to increased risk of a poor quality of life and is consistent with the univariate analysis in ***Supplementary Table 1***. As described in the main manuscript earlier social, fatigue, depression and emotional domains dominate the factor so these domains are more influential on the risk of a poor quality of life.
