# Supplementary material for: The inter‐relationship of symptom severity and quality of life in 2055 patients with primary biliary cholangitis
Source: Aliment Pharmacol Ther. 2016 Sep 19;44(10):1039–50. doi: 10.1111/apt.13794 (PMC5082554; doi:10.1111/apt.13794)
Supplement: Supplementary file 2 — Data S1. The UK‐PBC Consortium [file APT-44-1039-s002.docx]

Chin Lye Ch'ng ^1,2^, Mesbah Rahman ^3^, Tom Yapp ^4^, Richard Sturgess ^5^, Christopher Healey ^6^, Marek Czajkowski ^7,8,9^, Anton Gunasekera ^10,11^, Pranab Gyawali ^12^, Purushothaman Premchand ^13^, Kapil Kapur ^14^, Richard Marley ^15^, Graham Foster ^15^, Alan Watson ^16^, Aruna Dias ^17^, Javaid Subhani ^18^, Rory Harvey ^19^, Roger McCorry ^20^, David Ramanaden ^21^, Jaber Gasem ^22^, Richard Evans ^23^, Thiriloganathan Mathialahan ^24^, Christopher Shorrock ^25^, George Lipscomb ^26^, Paul Southern ^27^, Jeremy Tibble ^28,29^, David Gorard ^30,31,32^, Altaf Palegwala ^33^, Susan Jones ^34,35^, Marco Carbone ^36^, Mohamed Dawwas ^36^, Graeme Alexander ^36^, Sunil Dolwani ^37,38^, Martin Prince ^39^, Matthew Foxton ^40^, David Elphick ^41^, Harriet Mitchison ^42^, Ian Gooding ^43^, Mazn Karmo ^44^, Sushma Saksena ^45,46^, Mike Mendall ^47,48^, Minesh Patel ^49,50,51^, Roland Ede ^52,166^, Andrew Austin ^53^, Joanna Sayer ^54,55^, Lorraine Hankey ^56^, Christopher Hovell ^56^, Neil Fisher ^57^, Martyn Carter ^58,59^, Konrad Koss ^60^, Andrzej Piotrowicz ^61,62,63,64^, Charles Grimley ^65,66^, David Neal ^67,68^, Guan Lim ^69^, Sass Levi ^70,71^, Aftab Ala ^72^, Andrea Broad ^73^, Athar Saeed ^73^, Gordon Wood ^74^, Jonathan Brown ^75,76^, Mark Wilkinson ^77,78^, Harriet Gordon ^79^, John Ramage ^80^, Jo Ridpath ^81^, Theodore Ngatchu ^82,83,84^, Bob Grover ^85^, Syed Shaukat ^86^, Ray Shidrawi ^87^, George Abouda ^88,89^, Faiz Ali ^90^, Ian Rees ^91^, Imroz Salam ^92^, Mark Narain ^93^, Ashley Brown ^94,95,96^, Simon Taylor-Robinson ^96^, Simon Williams ^97^, Leonie Grellier ^98^, Paul Banim ^99^, Debashis Das ^100^, Andrew Chilton ^100^, Michael Heneghan ^101^, Howard Curtis ^102,103^, Markus Gess ^104^, Ian Drake ^105,106^, Mark Aldersley ^107,108^, Mervyn Davies ^107,108^, Rebecca Jones ^107,108^, Alastair McNair ^109^, Raj Srirajaskanthan ^110^, Maxton Pitcher ^111,112^, Sambit Sen ^113^, George Bird ^114,115^, Adrian Barnardo ^116^, Paul Kitchen ^116^, Kevin Yoong ^117^, Oza Chirag ^118,119^, Nurani Sivaramakrishnan ^120^, George MacFaul ^121^, David Jones ^122^, Amir Shah ^123^, Chris Evans ^124^, Subrata Saha ^125^, Katharine Pollock ^126,127^, Peter Bramley ^128,129^, Ashis Mukhopadhya ^130,131,132^, Andrew Fraser ^130,131,132^, Peter Mills ^133,134,135,136,137,138^, Christopher Shallcross ^139,140^, Stewart Campbell ^141,142,143^, Andrew Bathgate ^144,145,146^, Alan Shepherd ^147^, John Dillon ^148^, Simon Rushbrook ^149^, Robert Przemioslo ^150^, Christopher Macdonald ^151,152^, Jane Metcalf ^153,154^, Udi Shmueli ^155^, Andrew Davis ^156,157^, Asifabbas Naqvi ^158,159,160^, Tom Lee ^161,162^, Stephen D Ryder ^163,164^, Jane Collier ^165^, Howard Klass ^167,168,169,170^, Mary Ninkovic ^171,172^, Matthew Cramp ^173^, Nicholas Sharer ^174^, Richard Aspinall ^175^, Patrick Goggin ^175^, Deb Ghosh ^176,177^, Andrew Douds ^178^, Barbara Hoeroldt ^179^, Jonathan Booth ^180^, Earl Williams ^181^, Hyder Hussaini ^182^, William Stableforth ^182^, Reuben Ayres ^183^, Douglas Thorburn ^184^, Eileen Marshall ^184^, Andrew Burroughs ^184^, Steven Mann ^185,186^, Martin Lombard ^187^, Paul Richardson ^187^, Imran Patanwala ^187^, Julia Maltby ^188^, Matthew Brookes ^189^, Ray Mathew ^190,229^, Samir Vyas ^191^, Saket Singhal ^192^, Dermot Gleeson ^193,194^, Sharat Misra ^195,196^, Jeff Butterworth ^197,198^, Keith George ^199^, Tim Harding ^200,201^, Andrew Douglass ^202,203^, Simon Panter ^204^, Jeremy Shearman ^205^, Gary Bray ^206^, Graham Butcher ^207,208^, Daniel Forton ^209^, John Mclindon ^210,211^, Debashis Das ^212^, Matthew Cowan ^213^, Gregory Whatley ^214^, Aditya Mandal ^215,216^, Hemant Gupta ^215,216^, Pradeep Sanghi ^215,216^, Sanjiv Jain ^217^, Steve Pereira ^218^, Geeta Prasad ^219^, Gill Watts ^219^, Mark Wright ^220^, James Neuberger ^221^, Fiona Gordon ^222^, Esther Unitt ^223^, Allister Grant ^224,225,226^, Toby Delahooke ^224,225,226^, Andrew Higham ^227^, Alison Brind ^228^, Mark Cox ^230^, Subramaniam Ramakrishnan ^231^, Alistair King ^232,233,234^, Carole Collins ^235^, Simon Whalley ^236,237^, Andy Li ^238^, Jocelyn Fraser ^239^, Andrew Bell ^240^, Voi Shim Wong ^241^, Amit Singhal ^242,243^, Ian Gee ^244,245,246^, Yeng Ang ^247^, Rupert Ransford ^248^, James Gotto ^249^, Charles Millson ^250,251,252^, Jane Bowles ^253^, Caradog Thomas ^1,2,3,4,91,92^, Melanie Harrison ^5^, Roman Galaska ^6^, Jennie Kendall ^7,8,9^, Jessica Whiteman ^7,8,9^, Caroline Lawlor ^10,11^, Catherine Gray ^10,11^, Keith Elliott ^14^, Caroline Mulvaney-Jones ^21,22,23,24^, Lucie Hobson ^21,22,23,24^, Greta Van Duyvenvoorde ^25^, Alison Loftus ^26^, Katie Seward ^27^, Ruth Penn ^30,31,32^, Jane Maiden ^33^, Rose Damant ^33^, Janeane Hails ^36^, Rebecca Cloudsdale ^37,38^, Valeria Silvestre ^40^, Sue Glenn ^41^, Eleanor Dungca ^42^, Natalie Wheatley ^43^, Helen Doyle ^44^, Melanie Kent ^45,46^, Caroline Hamilton ^49,50,51^, Delyth Braim ^49,50,51^, Helen Wooldridge ^52^, Rachel Abrahams ^52^, Alison Paton ^53^, Nicola Lancaster ^54,55^, Andrew Gibbins ^56^, Karen Hogben ^56^, Phillipa Desousa ^58,59^, Florin Muscariu ^63,64^, Janine Musselwhite ^63,64^, Alexandra McKay ^66^, LaiTing Tan ^69^, Carole Foale ^72^, Jacqueline Brighton ^72^, Kerry Flahive ^74^, Estelle Nambela ^75,76^, Paula Townshend ^75,76^, Chris Ford ^75,76^, Sophie Holder ^75,76^, Caroline Palmer ^79,80^, James Featherstone ^81^, Mariam Nasseri ^85^, Joy Sadeghian ^87^, Bronwen Williams ^88,89^, Carol Thomas ^90^, Sally-Ann Rolls ^90^, Abigail Hynes ^93^, Claire Duggan ^93^, Sarah Jones ^93^, Mary Crossey ^94,95,96^, Glynis Stansfield ^97^, Carolyn MacNicol ^97^, Joy Wilkins ^98^, Elva Wilhelmsen ^99^, Parizade Raymode ^100^, Hye-Jeong Lee ^101^, Emma Durant ^105,106^, Rebecca Bishop ^107,108^, Noma Ncube ^109,110^, Sherill Tripoli ^111,112^, Rebecca Casey ^114,115^, Caroline Cowley ^116^, Richard Miller ^117^, Kathryn Houghton ^122^, Samantha Ducker ^122^, Fiona Wright ^123^, Bridget Bird ^125^, Gwen Baxter ^125^, Janie Keggans ^125^, Maggie Hughes ^128,129^, Emma Grieve ^130^, Karin Young ^130^, D Williams ^131^, Kate Ocker ^133^, Frances Hines ^139,140^, Kirsty Martin ^144,146^, Caron Innes ^147,148^, Talal Valliani ^150^, Helen Fairlamb ^151,152^, Sarah Thornthwaite ^151,152^, Anne Eastick ^153,154^, Elizabeth Tanqueray ^155^, Jennifer Morrison ^156^, Becky Holbrook ^156^, Julie Browning ^158,160^, Kirsten Walker ^161,162^, Susan Congreave ^163,164^, Juliette Verheyden ^163,164^, Susan Slininger ^163,164^, Lizzie Stafford ^165^, Denise O'Donnell ^165^, Mark Ainsworth ^165^, Susan Lord ^166^, Linda Kent ^167,168,169,170^, Linda March ^173^, Christine Dickson ^174^, Diane Simpson ^174^, Beverley Longhurst ^175^, Maria Hayes ^175^, Ervin Shpuza ^176,177^, Nikki White ^176,177^, Sarah Besley ^179^, Sallyanne Pearson ^179^, Alice Wright ^180^, Linda Jones ^180^, Emma Gunter ^181^, Hannah Dewhurst ^181^, Anna Fouracres ^182^, Liz Farrington ^182^, Lyn Graves ^182^, Suzie Marriott ^183^, Marina Leoni ^184^, David Tyrer ^187^, Kate Martin ^187^, Lola Dali-kemmery ^188^, Victoria Lambourne ^188^, Marie Green ^189^, Dawn Sirdefield ^190,229^, Kelly Amor ^190^, Julie Colley ^192^, Bal Shinder ^192^, Jayne Jones ^194^, Marisa Mills ^194^, Mandy Carnahan ^197,198^, Natalie Taylor ^199^, Kerenza Boulton ^199^, Julie Tregonning ^202,203^, Carly Brown ^204^, Gayle Clifford ^204^, Emily Archer ^205^, Maria Hamilton ^207,208^, Janette Curtis ^212^, Tracey Shewan ^213^, Sue Walsh ^214^, Karen Warner ^215,216^, Kimberley Netherton ^217^, Mcdonald Mupudzi ^220^, Bridget Gunson ^221^, Jane Gitahi ^222^, Denise Gocher ^223^, Sally Batham ^224,225^, Hilary Pateman ^224,225^, Senayon Desmennu ^224,225^, Jill Conder ^227^, Darren Clement ^228^, Susan Gallagher ^228^, Jacky Orpe ^229^, PuiChing Chan ^231^, Lynn Currie ^232,233,234^, Lynn O’Donohoe ^232,233,234^, Metod Oblak ^235^, Lisa Morgan ^237^, Marie Quinn ^238^, Isobel Amey ^239^, Yolanda Baird ^239^, Donna Cotterill ^240^, Lourdes Cumlat ^241^, Louise Winter ^247^, Sandra Greer ^247^, Katie Spurdle ^249^, Joanna Allison ^249^, Simon Dyer ^250,251^, Helen Sweeting ^252^, Jean Kordula ^253^

1. Abertawe Bro Morgannwg University Health Board, Morriston Hospital, Heol Maes Eglwys, Morriston, Swansea SA6 6NL

2. Abertawe Bro Morgannwg University Health Board, Singleton Hospital, Sketty Lane, Sketty, Swansea SA2 8QA

3. Abertawe Bro Morgannwg University Health Board, Neath Port Talbot Hospital, Baglan Way, Port Talbot SA12 7BX

4. Abertawe Bro Morgannwg University Health Board, Princess of Wales Hospital, Coity Road, Bridgend CF31 1RQ

5. Aintree University Hospitals NHS Foundation Trust, Aintree University Hospital, Longmoor Lane, Liverpool L9 7AL

6. Airedale NHS Foundation Trust, Airedale General Hospital, Skipton Road, Steeton, Keighley BD20 6TD

7. Aneurin Bevan University Health Board, Nevill Hall Hospital, Brecon Road, Abergavenny NP7 7EG

8. Aneurin Bevan University Health Board, Royal Gwent Hospital, Cardiff Road, Newport NP20 2UB

9. Aneurin Bevan University Health Board, Ysbyty Ystrad Fawr, Ystrad Fawr Way, Ystrad Mynach, Hengoed CF82 7EP

10. Ashford & St Peter's Hospitals NHS Foundation Trust, Ashford Hospital, London Road, Ashford TW15 3AA

11. Ashford & St Peter's Hospitals NHS Foundation Trust, St Peter's Hospital, Guildford Road, Chertsey KT16 0PZ

12. Barking, Havering and Redbridge University Hospitals NHS Trust, King George Hospital, Barley Lane, Ilford IG3 8YB

13. Barking, Havering and Redbridge University Hospitals NHS Trust, Queen's Hospital, Rom Valley Way, Romford RM7 0AG

14. Barnsley Hospital NHS Foundation Trust, Barnsley Hospital, Gawber Road, Barnsley S75 2EP

15. Barts Health NHS Trust, The Royal London Hospital, Whitechapel Road, Whitechapel, London E1 1BB

16. Barts Health NHS Trust, Whipps Cross University Hospital, Whipps Cross Road, Leytonstone, London E11 1NR

17. Barts Health NHS Trust, Newham University Hospital, Glen Road, Plaistow, London E13 8SL

18. Basildon and Thurrock University Hospitals NHS Foundation Trust, Basildon University Hospital, Nethermayne, Basildon SS16 5NL

19. Bedford Hospital NHS Trust, Bedford Hospital, Kempston Road, Bedford MK42 9DJ

20. Belfast Health and Social Care Trust, Royal Victoria Hospital, 274 Grosvenor Road, Belfast BT12 6BA

21. Betsi Cadwaladr University Health Board, Glan Clwyd Hospital, Rhyl LL18 5UJ

22. Betsi Cadwaladr University Health Board, Ysbyty Gwynedd, Penrhosgarnedd, Bangor LL57 2PW

23. Betsi Cadwaladr University Health Board, Llandudno General Hospital, Hospital Road, Llandudno LL30 1LB

24. Betsi Cadwaladr University Health Board, Wrexham Maelor Hospital, Croesnewydd Road, Wrexham LL13 7TD

25. Blackpool Teaching Hospitals NHS Foundation Trusts, Blackpool Victoria Hospital, Whinney Heys Road, Blackpool FY3 8NR

26. Bolton NHS Foundation Trust, Royal Bolton Hospital, Minerva Road, Farnworth, Bolton BL4 0JR

27. Bradford Teaching Hospitals NHS Foundation Trust, Bradford Royal Infirmary, Duckworth Lane, Bradford BD9 6RJ

28. Brighton and Sussex University Hospitals NHS Trust, Princess Royal Hospital, Lewes Road, Haywards Heath RH16 4EX

29. Brighton and Sussex University Hospitals NHS Trust, Royal Sussex County Hospital, Eastern Road, Brighton BN2 5BE

30. Buckinghamshire Healthcare NHS Trust, Amersham Hospital, Whielden Street, Amersham HP7 0JD

31. Buckinghamshire Healthcare NHS Trust, Stoke Mandeville Hospital, Mandeville Road, Aylesbury HP21 8AL

32. Buckinghamshire Healthcare NHS Trust, Wycombe Hospital, Queen Alexandra Road, High Wycombe HP11 2TT

33. Burton Hospitals NHS Foundation Trust, Queen's Hospital, Belvedere Road, Burton upon Trent DE13 0RB

34. Calderdale And Huddersfield NHS Foundation Trust, Calderdale Royal Hospital, Salterhebble, Halifax HX3 0PW

35. Calderdale And Huddersfield NHS Foundation Trust, Huddersfield Royal Infirmary, Acre Street, Lindley, Huddersfield HD3 3EA

36. Cambridge University Hospitals NHS Foundation Trust, Addenbrooke's Hospital, Cambridge Biomedical Campus, Hills Road, Cambridge CB2 0QQ

37. Cardiff and Vale University Health Board, University Hospital Llandough, Penlan Road, Llandough, Penarth CF64 2XX

38. Cardiff and Vale University Health Board, University Hospital of Wales, Heath Park, Cardiff CF14 4XW

39. Central Manchester University Hospitals NHS Foundation Trust, Manchester Royal Infirmary, Oxford Road, Manchester M13 9WL

40. Chelsea and Westminster Hospital NHS Foundation Trust, Chelsea and Westminster Hospital, 369 Fulham Road, London SW10 9NH

41. Chesterfield Royal Hospital NHS Foundation Trust, Chesterfield Royal Hospital, Calow, Chesterfield S44 5BL

42. City Hospitals Sunderland NHS Foundation Trust, Sunderland Royal Hospital, Kayll Road, Sunderland SR4 7TP

43. Colchester Hospital University NHS Foundation Trust, Colchester General Hospital, Turner Road, Colchester CO4 5JL

44. Countess of Chester Hospital NHS Foundation Trust, Countess of Chester Hospital, Countess of Chester Health Park, Liverpool Road, Chester CH2 1UL

45. County Durham and Darlington NHS Foundation Trust, Darlington Memorial Hospital, Hollyhurst Road, Darlington DL3 6HX

46. County Durham and Darlington NHS Foundation Trust, University Hospital of North Durham, North Road, Durham DH1 5TW

47. Croydon Health Services NHS Trust, Croydon University Hospital, 530 London Road, Croydon CR7 7YE

48. Croydon Health Services NHS Trust, Purley War Memorial Hospital, 856 Brighton Road, Purley CR8 2YL

49. Cwm Taf University Health Board, Prince Charles Hospital, Gurnos, Merthyr Tydfil CF47 9DT

50. Cwm Taf University Health Board, Royal Glamorgan Hospital, Ynysmaerdy, Llantrisant, Pontyclun CF72 8XR

51. Cwm Taf University Health Board, Ysbyty Cwm Cynon, New Road, Mountain Ash, Rhondda Cynon Taff CF45 4BZ

52. Dartford And Gravesham NHS Trust, Darent Valley Hospital, Darenth Wood Road, Dartford DA2 8DA

53. Derby Hospitals NHS Foundation Trust, Royal Derby Hospital, Uttoxeter Road, Derby DE22 3NE

54. Doncaster and Bassetlaw Hospitals NHS Foundation Trust, Bassetlaw Hospital, Blyth Road, Worksop S81 0BD

55. Doncaster and Bassetlaw Hospitals NHS Foundation Trust, Doncaster Royal Infirmary, Armthorpe Road, Doncaster DN2 5LT

56. Dorset County Hospitals NHS Foundation Trust, Dorset County Hospital, Williams Avenue, Dorchester DT1 2JY

57. Dudley Group of Hospitals NHS Trust, Russells Hall Hospital, Pensnett Road, Dudley DY1 2HQ

58. East and North Hertfordshire NHS Trust, Lister Hospital, Coreys Mill Lane, Stevenage SG1 4AB

59. East and North Hertfordshire NHS Trust, Queen Elizabeth II Hospital, Howlands, Welwyn Garden City AL7 4HQ

60. East Cheshire NHS Trust, Macclesfield District General Hospital, Victoria Road, Macclesfield SK10 3BL

61. East Kent Hospitals University NHS Foundation Trust, Buckland Hospital, Coombe Valley Road, Dover CT17 0HD

62. East Kent Hospitals University NHS Foundation Trust, Kent and Canterbury Hospital, Ethelbert Road, Canterbury CT1 3NG

63. East Kent Hospitals University NHS Foundation Trust, Queen Elizabeth The Queen Mother Hospital, St Peters Road, Margate CT9 4AN

64. East Kent Hospitals University NHS Foundation Trust, William Harvey Hospital Ashford, Kennington Road, Willesborough, Ashford TN24 0LZ

65. East Lancashire Hospitals NHS Trust, Burnley General Hospital, Casterton Avenue, Burnley BB10 2PQ

66. East Lancashire Hospitals NHS Trust, Royal Blackburn Hospital, Haslingden Road, Blackburn BB2 3HH

67. East Sussex Healthcare NHS Trust, Conquest Hospital, The Ridge, St Leonards-on-Sea TN37 7RD

68. East Sussex Healthcare NHS Trust, Eastbourne District General Hospital, Kings Drive, Eastbourne BN21 2UD

69. Epsom and St Helier University Hospitals NHS Trust, Epsom General Hospital, Epsom Hospital, Dorking Road, Epsom KT18 7EG

70. Frimley Health NHS Foundation Trust, Heatherwood Hospital, London Road, Ascot SL5 8AA

71. Frimley Health NHS Foundation Trust, Wexham Park Hospital, Wexham, Slough SL2 4HL

72. Frimley Health NHS Foundation Trust, Frimley Park Hospital, Portsmouth Road, Frimley GU16 7UJ

73. Gateshead Health NHS Foundation Trust, Queen Elizabeth Hospital, Sheriff Hill, Gateshead NE9 6SX

74. George Eliot Hospital NHS Trust, George Eliot Hospital, Eliot Way, Nuneaton CV10 7DJ

75. Gloucestershire Hospitals NHS Foundation Trust, Cheltenham General Hospital, Sandford Road, Cheltenham GL53 7AN

76. Gloucestershire Hospitals NHS Foundation Trust, Gloucestershire Royal Hospital, Great Western Road, Gloucester GL1 3NN

77. Guy's and St Thomas' NHS Foundation Trust, Guy's Hospital, Great Maze Pond, London SE1 9RT

78. Guy's and St Thomas' NHS Foundation Trust, St Thomas' Hospital, Westminster Bridge Road, London SE1 7EH

79. Hampshire Hospitals NHS Foundation Trust, Royal Hampshire County Hospital, Romsey Road, Winchester SO22 5DG

80. Hampshire Hospitals NHS Foundation Trust, Basingstoke and North Hampshire Hospital, Aldermaston Road, Basingstoke RG24 9NA

81. Harrogate and District NHS Foundation Trust, Harrogate District Hospital, Lancaster Park Road, Harrogate HG2 7SX

82. Heart of England NHS Foundation Trust, Good Hope Hospital, Rectory Road, Sutton Coldfield, Birmingham B75 7RR

83. Heart of England NHS Foundation Trust, Heartlands Hospital, Bordesley Green East, Birmingham B9 5SS

84. Heart of England NHS Foundation Trust, Solihull Hospital, Lode Lane, Solihull B91 2JL

85. Hillingdon Hospitals NHS Foundation Trust, Hillingdon Hospital, Pield Heath Road, Uxbridge UB8 3NN

86. Hinchingbrooke Health Care NHS Trust, Hinchingbrooke Hospital, Hinchingbrooke Park, Huntingdon PE29 6NT

87. Homerton University Hospital NHS Foundation Trust, Homerton University Hospital, Homerton Row, London E9 6SR

88. Hull And East Yorkshire Hospitals NHS Trust, Castle Hill Hospital, Castle Road, Cottingham HU16 5JQ

89. Hull And East Yorkshire Hospitals NHS Trust, Hull Royal Infirmary, Anlaby Road, Hull HU3 2JZ

90. Hywel Dda University Health Board, Withybush General Hospital, Fishguard Road, Haverfordwest SA61 2PZ

91. Hywel Dda University Health Board, Prince Philip Hospital, Bryngwyn Mawr, Dafen, Llanelli SA14 8QF

92. Hywel Dda University Health Board, Glangwili General Hospital, Dolgwilli Road, Carmarthen SA31 2AF

93. Hywel Dda University Health Board, Bronglais Hospital, Caradog Road, Aberystwyth SY23 1ER

94. Imperial College Healthcare NHS Trust, Charing Cross Hospital, Fulham Palace Road, London W6 8RF

95. Imperial College Healthcare NHS Trust, Hammersmith Hospital, Du Cane Road, London W12 0HS

96. Imperial College Healthcare NHS Trust, St Mary's Hospital, Praed Street, London W2 1NY

97. Ipswich Hospital NHS Trust, Ipswich Hospital, Heath Road, Ipswich IP4 5PD

98. Isle of Wight NHS Trust, St Mary's Hospital, Parkhurst Road, Newport PO30 5TG

99. James Paget University Hospitals NHS Foundation Trust, James Paget Hospital, Lowestoft Road, Gorleston, Great Yarmouth NR31 6LA

100. Kettering General Hospital NHS Foundation Trust, Kettering General Hospital, Rothwell Road, Kettering NN16 8UZ

101. Kings College Hospital NHS Foundation Trust, King's College Hospital, Denmark Hill, London SE5 9RS

102. King's College Hospital NHS Foundation Trust, Beckenham Beacon, 395 Croydon Road, Beckenham BR3 3QL

103. King's College Hospital NHS Foundation Trust, Princess Royal University Hospital, Farnborough Common, Orpington BR6 8ND

104. Kingston Hospital NHS Foundation Trust, Kingston Hospital, Galsworthy Road, Kingston upon Thames KT2 7QB

105. Lancashire Teaching Hospitals NHS Foundation Trust, Chorley and South Ribble Hospital, Preston Road, Chorley PR7 1PP

106. Lancashire Teaching Hospitals NHS Foundation Trust, Royal Preston Hospital, Sharoe Green Lane North, Preston PR2 9HT

107. Leeds Teaching Hospitals NHS Trust, Leeds General Infirmary, Great George Street, Leeds LS1 3EX

108. Leeds Teaching Hospitals NHS Trust, St James's University Hospital, Beckett Street, Leeds LS9 7TF

109. Lewisham and Greenwich NHS Trust, The Queen Elizabeth, Woolwich, Stadium Road, Greenwich SE18 4QH

110. Lewisham and Greenwich NHS Trust, Lewisham Hospital, High Street, Lewisham SE13 6LH

111. London North West Healthcare NHS Trust, Central Middlesex Hospital, Acton Lane, Park Royal, London NW10 7NS

112. London North West Healthcare NHS Trust, Northwick Park and St Mark's Hospitals, Watford Road, Harrow HA1 3UJ

113. Luton and Dunstable University Hospital NHS Foundation Trust, Luton and Dunstable University Hospital, Lewsey Road, Luton LU4 0DZ

114. Maidstone and Tunbridge Wells NHS Trust, Maidstone Hospital, Hermitage Lane, Maidstone ME16 9QQ

115. Maidstone and Tunbridge Wells NHS Trust, Tunbridge Wells Hospital, Tonbridge Road, Pembury, Tunbridge Wells TN2 4QJ

116. Medway NHS Foundation Trust, Medway Maritime Hospital, Windmill Road, Gillingham ME7 5NY

117. Mid Cheshire Hospitals NHS Foundation Trust, Leighton Hospital, Middlewich Road, CW1 4QJ

118. Mid Essex Hospital Services NHS Trust, Broomfield Hospital, Court Road, Chelmsford CM1 7ET

119. Mid Essex Hospital Services NHS Trust, St Peters Hospital, Spital Road, Maldon CM9 6EG

120. Mid Yorkshire Hospitals NHS Trust, Dewsbury and District Hospital, Halifax Road, Dewsbury WF13 4HS

121. Milton Keynes Hospital NHS Foundation Trust, Milton Keynes Hospital, Standing Way, Milton Keynes MK6 5LD

122. Newcastle upon Tyne Hospitals NHS Foundation Trust, Freeman Hospital, Freeman Road, High Heaton, Newcastle upon Tyne NE7 7DN

123. NHS Ayrshire & Arran, University Hospital Crosshouse, Kilmarnock Road, Kilmarnock KA2 0BE

124. NHS Borders, Borders General Hospital,, Melrose TD6 9BS

125. NHS Dumfries & Galloway, Dumfries and Galloway Royal Infirmary, Bankend Road, Dumfries DG1 4AP

126. NHS Fife, Queen Margaret Hospital, Whitefield Road, Dunfermline KY12 0SU

127. NHS Fife, Victoria Hospital, Hayfield Road, Kirkcaldy KY2 5AH

128. NHS Forth Valley, Forth Valley Royal Hospital, Stirling Road, Larbert FK5 4WR

129. NHS Forth Valley, Stirling Community Hospital, Livilands, Stirling FK8 2AU

130. NHS Grampian, Aberdeen Royal Infirmary, Foresterhill, Aberdeen AB25 2ZN

131. NHS Grampian, Dr Gray's Hospital, Elgin IV30 1SN

132. NHS Grampian, Woolmanhill Hospital, Skene Street, Aberdeen AB25 1LD

133. NHS Greater Glasgow and Clyde, Gartnavel General Hospital, 1053 Great Western Road, Glasgow G12 0YN

134. NHS Greater Glasgow and Clyde, Glasgow Royal Infirmary, 84 Castle Street, Glasgow G4 0SF

135. NHS Greater Glasgow and Clyde, Inverclyde Royal Hospital, Larkfield Road, Greenock PA16 0XN

136. NHS Greater Glasgow and Clyde, Royal Alexandra Hospital, Corsebar Road, Paisley PA2 9PN

137. NHS Greater Glasgow and Clyde, Southern General Hospital, 1345 Govan Road, Glasgow G51 4TF

138. NHS Greater Glasgow and Clyde, Victoria Infirmary, Langside Road, Glasgow G42 9TY

139. NHS Highland, Caithness General Hospital, Bankhead Road, Wick KW1 5NS

140. NHS Highland, Raigmore Hospital, Old Perth Road, Inverness IV2 3UJ

141. NHS Lanarkshire, Hairmyres Hospital, Eaglesham Road, East Kilbride G75 8RG

142. NHS Lanarkshire, Monklands Hospital, Monkscourt Avenue, Airdrie ML6 0JS

143. NHS Lanarkshire, Wishaw General Hospital, 50 Netherton Street, Wishaw ML2 0DP

144. NHS Lothian, Royal Infirmary of Edinburgh, 51 Little France Crescent, Old Dalkeith Road, Edinburgh EH16 4SA

145. NHS Lothian, St John's Hospital, Howden Road West, Howden, Livingston EH54 6PP

146. NHS Lothian, Western General Hospital, Crewe Road South, Edinburgh EH4 2XU

147. NHS Tayside, Perth Royal Infirmary, Taymount Terrace, Perth PH1 1NX

148. NHS Tayside, Ninewells Hospital,, Dundee DD1 9SY

149. Norfok and Norwich University Hospitals NHS Foundation Trust, Norfolk and Norwich University Hospital, Colney Lane, Norwich NR4 7UY

150. North Bristol NHS Trust, Frenchay Hospital, Frenchay Park Road, Bristol BS16 1LE

151. North Cumbria University Hospitals NHS Foundation Trust, Cumberland Infirmary, Newtown Road, Carlisle CA2 7HY

152. North Cumbria University Hospitals NHS Foundation Trust, West Cumberland Hospital, Hensingham, Whitehaven CA28 8JG

153. North Tees and Hartlepool NHS Foundation Trust, University Hospital of Hartlepool, Holdforth Road, Hartlepool TS24 9AH

154. North Tees and Hartlepool NHS Foundation Trust, University Hospital of North Tees, Hardwick, Stockton on Tees TS19 8PE

155. Northampton General Hospital NHS Trust, Northampton General Hospital, Cliftonville, Northampton NN1 5BD

156. Northern Devon Healthcare NHS Trust, North Devon District Hospital, Raleigh Park, Barnstaple EX31 4JB

157. Northern Health and Social Care Trust, Whiteabbey Hospital, Doagh Road, Newtownabbey BT37 9RH

158. Northern Lincolnshire and Goole NHS Foundation Trust, Diana, Princess of Wales Hospital, Scartho Road, Grimsby DN33 2BA

159. Northern Lincolnshire and Goole NHS Foundation Trust, Goole and District Hospital, Woodland Avenue, Goole DN14 6RX

160. Northern Lincolnshire and Goole NHS Foundation Trust, Scunthorpe General Hospital, Cliff Gardens, Scunthorpe DN15 7BH

161. Northumbria Healthcare NHS Foundation Trust, Hexham General Hospital, Corbridge Road, Hexham NE46 1QJ

162. Northumbria Healthcare NHS Foundation Trust, North Tyneside Hospital, Rake Lane, North Shields NE29 8NH

163. Nottingham University Hospitals NHS Trust, Nottingham City Hospital, Hucknall Road, Nottingham NG5 1PB

164. Nottingham University Hospitals NHS Trust, Queen's Medical Centre, Derby Road, Nottingham NG7 2UH

165. Oxford University Hospitals NHS Trust, John Radcliffe Hospital, Headley Way, Headington, Oxford OX3 9DU

166. Oxleas NHS Foundation Trust, Queen Mary's Hospital Sidcup, Frognal Avenue, Sidcup DA14 6LT

167. Pennine Acute Hospitals NHS Trust, Fairfield General Hospital, Rochdale Old Road, Bury BL9 7TD

168. Pennine Acute Hospitals NHS Trust, North Manchester General Hospital, Delaunays Road, Crumpsall M8 5RB

169. Pennine Acute Hospitals NHS Trust, Rochdale Infirmary, Whitehall Street, Rochdale OL12 0NB

170. Pennine Acute Hospitals NHS Trust, The Royal Oldham Hospital, Rochdale Road, Oldham OL1 2JH

171. Peterborough and Stamford Hospitals NHS Foundation Trust, Peterborough City Hospital, Edith Cavell Campus, Bretton Gate, Peterborough PE3 9GZ

172. Peterborough and Stamford Hospitals NHS Foundation Trust, Stamford & Rutland Hospital, Ryhall Road, Stamford PE9 1UA

173. Plymouth Hospitals NHS Trust, Derriford Hospital, Derriford Road, Plymouth PL6 8DH

174. Poole Hospital NHS Foundation Trust, Poole Hospital, Longfleet Road, Poole BH15 2JB

175. Portsmouth Hospitals NHS Trust, Queen Alexandra Hospital, Cosham, Portsmouth PO6 3LY

176. Princess Alexandra Hospital NHS Trust, St Margaret's Hospital, The Plain, Epping CM16 6TN

177. Princess Alexandra Hospital NHS Trust, The Princess Alexandra Hospital, Hamstel Road, Harlow CM20 1QX

178. Queen Elizabeth Hospital King's Lynn NHS Foundation Trust, The Queen Elizabeth Hospital King's Lynn, Gayton Road, King's Lynn PE30 4ET

179. Rotherham NHS Foundation Trust, Rotherham Hospital, Moorgate Road, Rotherham S60 2UD

180. Royal Berkshire NHS Foundation Trust, Royal Berkshire Hospital, Craven Road, Reading RG1 5AN

181. Royal Bournemouth and Christchurch Hospitals NHS Foundation Trust, Royal Bournemouth Hospital, Castle Lane East, Bournemouth BH7 7DW

182. Royal Cornwall Hospitals NHS Trust, Royal Cornwall Hospital, Treliske, Truro TR1 3LJ

183. Royal Devon and Exeter NHS Foundation Trust, Royal Devon and Exeter Hospital, Barrack Road, Exeter EX2 5DW

184. Royal Free London NHS Foundation Trust, The Royal Free Hospital, Pond Street, London NW3 2QG

185. Royal Free London NHS Foundation Trust, Barnet Hospital, Wellhouse Lane, Barnet EN5 3DJ

186. Royal Free London NHS Foundation Trust, Chase Farm Hospital, The Ridgeway, Enfield EN2 8JL

187. Royal Liverpool and Broadgreen University Hospitals NHS Trust, Royal Liverpool University Hospital, Prescot Street, Liverpool L7 8XP

188. Royal United Hospitals Bath NHS Foundation Trust, Royal United Bath Hospital, Combe Park, Bath BA1 3NG

189. Royal Wolverhampton Hospitals NHS Trust, New Cross Hospital, Wolverhampton Road, Wolverhampton WV10 0QP

190. Royal Wolverhampton Hospitals NHS Trust, Cannock Chase Hospital, Brunswick Road, Cannock WS11 5XY

191. Salisbury NHS Foundation Trust, Salisbury District Hospital,, Salisbury SP2 8BJ

192. Sandwell and West Birmingham Hospitals NHS Trust, Sandwell General Hospital, Lyndon, West Bromwich B71 4HJ

193. Sheffield Teaching Hospitals NHS Foundation Trust, Northern General Hospital, Herries Road, Sheffield S5 7AU

194. Sheffield Teaching Hospitals NHS Foundation Trust, Royal Hallamshire Hospital, Glossop Road, Sheffield S10 2JF

195. Sherwood Forest Hospitals NHS Foundation Trust, King's Mill Hospital, Mansfield Road, Sutton in Ashfield NG17 4JL

196. Sherwood Forest Hospitals NHS Foundation Trust, Newark Hospital, Boundary Road, Newark NG24 4DE

197. Shrewsbury and Telford Hospital NHS Trust, Princess Royal Hospital, Apley Castle, Telford TF1 6TF

198. Shrewsbury and Telford Hospital NHS Trust, Royal Shrewsbury Hospital, Mytton Oak Road, Shrewsbury SY3 8XQ

199. South Devon Healthcare NHS Foundation Trust, Torbay Hospital, Lowes Bridge, Torquay TQ2 7AA

200. South Eastern Health and Social Care Trust, Lagan Valley Hospital, 39 Hillsborough Road, Lisburn BT28 1JP

201. South Eastern Health and Social Care Trust, Ulster Hospital, Upper Newtownards Road, Dundonald, Belfast BT16 1RH

202. South Tees Hospitals NHS Foundation Trust, The James Cook University Hospital, Marton Road, Middlesbrough TS4 3BW

203. South Tees Hospitals NHS Foundation Trust, Friarage Hospital, Northallerton DL6 1JG

204. South Tyneside NHS Foundation Trust, South Tyneside District Hospital, Harton Lane, South Shields NE34 0PL

205. South Warwickshire NHS Foundation Trust, Warwick Hospital, Lakin Road, Warwick CV34 5BW

206. Southend University Hospital NHS Foundation Trust, Southend Hospital, Prittlewell Chase, Westcliff-on-Sea SS0 0RY

207. Southport & Ormskirk Hospital NHS Trust, Ormskirk District General Hospital, Wigan Road, Ormskirk L39 2AZ

208. Southport & Ormskirk Hospital NHS Trust, Southport and Formby District General Hospital, Town Lane, Kew, Southport PR8 6PN

209. St George’s University Hospitals NHS Foundation Trust, St George's Hospital, Blackshaw Road, Tooting, London SW17 0QT

210. St Helens and Knowsley Teaching Hospitals NHS Trust, St Helens Hospital, Marshalls Cross Road, St Helens WA9 3DA

211. St Helens and Knowsley Teaching Hospitals NHS Trust, Whiston Hospital, Warrington Road, Prescot L35 5DR

212. Stockport NHS Foundation Trust, Stepping Hill Hospital, Poplar Grove, Hazel Grove, Stockport SK2 7JE

213. Surrey and Sussex Healthcare NHS Trust, East Surrey Hospital, Canada Avenue, Redhill RH1 5RH

214. Tameside Hospital NHS Foundation Trust, Tameside General Hospital, Fountain Street, Ashton-under-Lyne OL6 9RW

215. United Lincolnshire Hospitals NHS Trust, Lincoln County Hospital, Greetwell Road, Lincoln LN2 5QY

216. United Lincolnshire Hospitals NHS Trust, Grantham and District Hospital, 101 Manthorpe Road, Grantham NG31 8DG

217. United Lincolnshire Hospitals NHS Trust, Pilgrim Hospital Boston, Sibsey Road, Boston PE21 9QS

218. University College London Hospitals NHS Foundation Trust, University College Hospital, 235 Euston Road, London NW1 2BU

219. University Hospital of South Manchester NHS Foundation Trust, Wythenshawe Hospital, Southmoor Road, Wythenshawe, Manchester M23 9LT

220. University Hospital Southampton NHS Foundation Trust, Southampton General Hospital, Tremona Road, Southampton SO16 6YD

221. University Hospitals Birmingham NHS Foundation Trust, Queen Elizabeth Hospital , Mindelsohn Way, Edgbaston, Birmingham B15 2GW

222. University Hospitals Bristol NHS Foundation Trust, Bristol Royal Infirmary, Upper Maudlin Street, Bristol BS2 8HW

223. University Hospitals Coventry and Warwickshire NHS Trust, University Hospital, Clifford Bridge Road, Coventry CV2 2DX

224. University Hospitals of Leicester NHS Trust, Glenfield Hospital, Groby Road, Leicester LE3 9QP

225. University Hospitals of Leicester NHS Trust, Leicester General Hospital, Gwendolen Road, Leicester LE5 4PW

226. University Hospitals of Leicester NHS Trust, Leicester Royal Infirmary, Infirmary Square, Leicester LE1 5WW

227. University Hospitals of Morecambe Bay NHS Foundation Trust, Royal Lancaster Infirmary, Ashton Road, Lancaster LA1 4RP

228. University Hospitals of North Midlands NHS Trust, Royal Stoke University Hospital, Newcastle Road, Stoke-on-Trent ST4 6QG

229. University Hospitals of North Midlands NHS Trust, County Hospital, Weston Road, Stafford ST16 3SA

230. Walsall Healthcare NHS Trust, Walsall Manor Hospital, Moat Road, Walsall WS2 9PS

231. Warrington and Halton Hospitals NHS Foundation Trust, Warrington Hospital, Lovely Lane, Warrington WA5 1QG

232. West Hertfordshire Hospitals NHS Trust, Hemel Hempstead General Hospital, Hillfield Road, Hemel Hempstead HP2 4AD

233. West Hertfordshire Hospitals NHS Trust, St Albans City Hospital, Waverley Road, St Albans AL3 5PN

234. West Hertfordshire Hospitals NHS Trust, Watford General Hospital, Vicarage Road, Watford WD18 0HB

235. West Middlesex University NHS Trust, West Middlesex University Hospital, Twickenham Road, Isleworth TW7 6AF

236. West Suffolk NHS Foundation Trust, Walnut Tree Hospital, Walnut Tree Lane, Sudbury CO10 1BE

237. West Suffolk NHS Foundation Trust, West Suffolk Hospital, Hardwick Lane, Bury St Edmunds IP33 2QZ

238. Western Sussex Hospitals NHS Foundation Trust, Worthing Hospital, Lyndhurst Road, Worthing BN11 2DH

239. Western Sussex Hospitals NHS Foundation Trust, St Richard's Hospital, Spitalfield Lane, Chichester PO19 6SE

240. Weston Area Health NHS Trust, Weston General Hospital, Grange Road, Uphill, Weston super Mare BS23 4TQ

241. Whittington Hospital NHS Trust, The Whittington Hospital, Magdala Avenue, London N19 5NF

242. Wirral University Teaching Hospital NHS Foundation Trust, Arrowe Park Hospital, Upton CH49 5PE

243. Wirral University Teaching Hospital NHS Foundation Trust, Victoria Central Hospital, Mill Lane, Wallasey CH44 5UF

244. Worcestershire Acute Hospitals NHS Trust, Alexandra Hospital, Woodrow Drive, Redditch B98 7UB

245. Worcestershire Acute Hospitals NHS Trust, Kidderminster Hospital and Treatment Centre, Bewdley Road, Kidderminster DY11 6RJ

246. Worcestershire Acute Hospitals NHS Trust, Worcestershire Royal Hospital, Charles Hastings Way, Worcester WR5 1DD

247. Wrightington, Wigan And Leigh NHS Trust, Royal Albert Edward Infirmary, Wigan Lane, Wigan WN1 2NN

248. Wye Valley NHS Trust, The County Hospital, Stonebow Road, Hereford HR1 2BN

249. Yeovil District Hospital NHS Foundation Trust, Yeovil District Hospital, Higher Kingston, Yeovil BA21 4AT

250. York Teaching Hospital NHS Foundation Trust, Bridlington Hospital, Bessingby Road, Bridlington YO16 4QP

251. York Teaching Hospital NHS Foundation Trust, Scarborough Hospital, Woodlands Drive, Scarborough YO12 6QL

252. York Teaching Hospital NHS Foundation Trust, The York Hospital, Wigginton Road, York YO31 8HE

253. Great Western Hospitals NHS Foundation Trust, Marlborough Road, Swindon, Wiltshire SN3 6BB
